# Asymmetric microarray data produces gene lists highly predictive of research literature on multiple cancer types

**DOI:** 10.1186/1471-2105-11-483

**Published:** 2010-09-27

**Authors:** Noor B Dawany, Aydin Tozeren

**Affiliations:** 1 Center for Integrated Bioinformatics, Drexel University, Bossone Research Building 711, 3102 Market Street, Philadelphia, PA 19104, USA

## Abstract

**Background:**

Much of the public access cancer microarray data is asymmetric, belonging to datasets containing no samples from normal tissue. Asymmetric data cannot be used in standard meta-analysis approaches (such as the inverse variance method) to obtain large sample sizes for statistical power enrichment. Noting that plenty of normal tissue microarray samples exist in studies not involving cancer, we investigated the viability and accuracy of an integrated microarray analysis approach based on significance analysis of microarrays (merged SAM) using a collection of data from separate diseased and normal samples.

**Results:**

We focused on five solid cancer types (colon, kidney, liver, lung, and pancreas), where available microarray data allowed us to compare meta-analysis and integrated approaches. Our results from the merged SAM significantly overlapped gene lists from the validated inverse-variance method. Both meta-analysis and merged SAM approaches successfully captured the aberrances in the cell cycle that commonly occur in the different cancer types. However, the integrated SAM analysis replicated the known cancer literature (excluding microarray studies) with much more accuracy than the meta-analysis.

**Conclusion:**

The merged SAM test is a powerful, robust approach for combining data from similar platforms and for analyzing asymmetric datasets, including those with only normal or only cancer samples that cannot be utilized by meta-analysis methods. The integrated SAM approach can also be used in comparing global gene expression between various subtypes of cancer arising from the same tissue.

## Background

Microarray studies typically provide intensity levels for thousands of genes. However, not only are the individual datasets usually small in size, but the inferences made from individual studies are often inconsistent with similar studies [1]. As thousands of microarray samples have accumulated in publicly accessible databases in the last decade [2-4], several statistical methods have been developed to allow for the combination and comparison of data from multiple sources. Among the many methodologies that exist, which deal with combining different microarray datasets, are the permutation tests [5,6], parametric tests and clustering [7], rank-aggregation procedures [8,9], rank products [10], METRADISC [1], and inverse-variance [11-13]. The utilization of vast amounts of microarray data provided by different groups is considered to increase the reliability of the results and weakens the effects of lab-specific noise [14].

The meta-analysis procedures cited above combine results from different studies. Each dataset is analyzed separately. Genes are associated with an effect size or a p-value. These are then combined across all analyses and a top-ranked gene list is generated based on the aggregated effect size or p-value [15]. While some meta-analysis methods require the use of raw data [5,6,11], others can depend solely on the ranking of genes from various studies [8,9]. The meta-analysis is robust in the sense that it allows for comparisons across different platforms and analytical techniques (cDNA and oligonucleotide microarrays). However, the most important limitation the meta-analysis poses is that it requires datasets to include both control and test samples. Previous studies showed that aggregating data prior to obtaining results is usually more powerful than obtaining separate statistics from each dataset and then integrating the results [16]. Therefore, based on the grounds of previous studies that revealed the predictive potential of integrated microarray [17-19], we consider in this study a large-scale merge approach to the significance analysis of microarrays (SAM; [20]) test that can utilize asymmetric datasets. SAM was chosen as the significance test because it is extensively used in our lab and has previously been used in normal, tumor and cell line comparisons [21]. Its performance has been shown to be superior to that of other conventional microarray analysis methods. Moreover, SAM uses random iterations to calculate the false discovery rate, allowing the user to control and adjust results accordingly [20].

To test the performances of the meta-analysis and the merged SAM approach, we compiled microarray data from 31 laboratories, resulting in a database containing 339 healthy tissue samples and 1,429 cancer samples from 5 different tissue types using comparable Affymetrix platforms. The tumor tissue types considered in this study -colon, kidney, liver, lung, and pancreas - had multiple microarray datasets containing both normal and disease samples. The meta-analysis approach has already been employed by a few cancer microarray studies either focusing on a single tissue type [5,13,22-24] or across different tissues in order to identify gene sets associated with common cancer mechanisms [6,11,25]. For the purpose of this study, the inverse-variance (IV) test was adopted from the work of Ramasamy et al. [11] to compare the quality of our results, since it is believed to be the most comprehensive meta-analysis method for two-class microarray gene expression analyses. With this large-scale database we generated significantly altered gene lists for each individual tissue as well as across all five tissue types, using both the IV and the merged SAM tests. Our results show that the merged SAM analysis, when based on large-scale data, not only significantly overlaps the results produced by the IV meta-analysis, but also provides gene lists that replicate the known cancer literature at least as well as the IV test.

## Results

### Datasets and approaches

**Figure 1:**
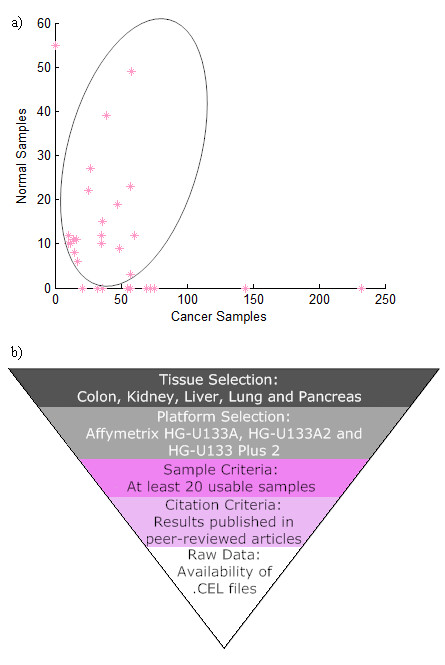
**Overview of Microarray Datasets Used and Dataset Inclusion Criteria**: a) Distribution of all Affymetrix microarray data used based on the number of cancer versus normal samples in each dataset. Datasets used for IV1/SAM1 test are shown inside the ellipse. Additional datasets included in SAM2 only are located on the axes. b) Selection method used for the inclusion of Affymetrix datasets used for the analyses in this study.

Three different groups of microarray datasets were used to evaluate (a) the intersection of significant gene lists predicted by meta-analysis and merged SAM methods and (b) compare these predictions with research literature excluding microarray studies. Group 1 is composed of Affymetrix microarray datasets containing both cancer and normal samples for five different cancer tissues (Table 1). The gene set predictions resulting from analysis of this data with the use of meta-analysis and merged SAM are denoted as IV1 and SAM1, respectively. Each dataset was analyzed separately for the IV1 test and a final gene list was produced based on the weighted results from the individual datasets. The SAM1 test was applied to the same Affymetrix data from each tissue after their merger, with all samples being normalized together, regardless of dataset. Group 2 of microarray datasets used in intersection analysis and literature comparison contained cDNA microarray datasets in addition to the Affymetrix data in Group 1. The gene lists predicted by meta-analysis using these datasets were called IV2. We used Group 2 to take full advantage of the capability of meta-analysis in integrating microarray datasets from different technologies. Group 3 contained asymmetric Affymetrix data in addition to data in Group 1 (Table 1). The gene list corresponding to Group 3 data predicted by merged SAM is referred to as SAM2. Figure 1a shows the overall characteristics of the Affymetrix datasets used in the analysis. The intersections of the predicted gene lists obtained with the two methods on the three different groups of datasets are summarized in Table 2. The table also presents the p-values corresponding to the intersections based on hypergeometric test.

**Table 1:** Overview of datasets used and distribution of microarray samples

Analysis	Tissue	Accession #	Normal	Cancer	Platform
**IV1/IV2/SAM1/SAM2**	Colon	E-MTAB-57	22	25	A
		GSE4107	10	12	P2
		GSE4183	8	15	P2
	Kidney	E-TABM-282	11	16	P2
		GSE11024†	12	60	P2
		GSE11151	3	57	P2
		GSE14762†	12	10	P2
		GSE15641	23	57	A
		GSE6344	10	10	A
		GSE7023	12	35	P2
	Liver	GSE14323	19	47	A/A2
		GSE6764	10	35	P2
	Lung	E-MEXP-231	9	49	A
		GSE10072	49	58	A
		GSE7670	27	27	A
	Pancreas	E-MEXP-1121†	6	17	A
		E-MEXP-950	11	14	A
		GSE15471	39	39	P2
		GSE16515	15	36	P2
		**Total:**	**294**	**619**	

**SAM2**	Colon	E-MEXP-1224	0	55	A
		E-MEXP-383	0	36	A
		E-TABM-176	55	0	P2
		GSE12945	0	36	A
		GSE17538	0	232	P2
	Kidney	GSE10320	0	144	A
		GSE11904	0	21	A2
	Liver	E-TABM-292	0	32	A
		E-TABM-36	0	57	A
		GSE9843	0	69	P2
	Lung	GSE10445	0	72	P2
		GSE12667	0	75	P2
		**Total:**	**55**	**829**	

**IV2**	Colon	GSE6988	28	52	cDNA
	Kidney	GSE3	81	90	cDNA
	Lung	GSE7367	24	24	cDNA
		GSE2088	30	57	cDNA
		GSE8596	6	69	cDNA
		**Total:**	**169**	**292**	

† Datasets included replicated samples

Platforms: A: HG-U133A, A2: HG-U133A2, P2: HG-U133 Plus 2

**Table 2:** The overlap among n top-ranked genes between the IV1 and SAM1/SAM2 tests with corresponding p-values of the intersection, as well as among the top 400 genes between the similar approaches (IV1/IV2 and SAM1/SAM2).

**IV1 **∩ **SAM1**												
n	Colon		Kidney		Liver		Lung		Pancreas		All	
	Overlap	P-Value	Overlap	P-Value	Overlap	P-Value	Overlap	P-Value	Overlap	P-Value	Overlap	P-Value
**10**	3	1.01E-07	0	0.989487	5	9.98E-14	2	4.49E-05	0	0.989487	0	0.989487
**50**	11	8.67E-16	5	5.71E-06	17	7.85E-28	14	1.44E-21	8	1.49E-10	6	2.05E-07
**100**	26	4.68E-30	23	3.04E-25	24	8.09E-27	34	4.21E-44	17	1.93E-16	18	7.94E-18
**200**	62	1.88E-57	68	1.57E-66	53	9.78E-45	93	2.56E-109	34	8.96E-22	64	2.00E-60
**300**	109	2.48E-91	106	4.38E-87	89	1.69E-64	146	5.40E-150	51	7.65E-24	103	6.46E-83
**400**	132*	3.74E-98	146	1.41E-104	119	7.44E-72	198	8.42E-181	71	2.66E-26	140	6.82E-97
												
**IV1 **∩ **SAM2**												
**n**	**Colon**		**Kidney**		**Liver**		**Lung**		**Pancreas**		**All**	
	**Overlap**	**P-Value**	**Overlap**	**P-Value**	**Overlap**	**P-Value**	**Overlap**	**P-Value**	**Overlap**	**P-Value**	**Overlap**	**P-Value**
**10**	3	1.01E-07	0	0.989487	4	1.31E-10	2	4.49E-05	0	0.989487	0	0.989487
**50**	12	1.17E-17	5	5.71E-06	12	1.17E-17	8	1.49E-10	8	1.49E-10	5	5.71E-06
**100**	32	1.97E-40	23	3.04E-25	24	8.09E-27	28	2.09E-33	17	1.93E-16	21	3.50E-22
**200**	67	5.51E-65	66	1.88E-63	43	5.97E-32	69	4.34E-68	34	8.96E-22	65	6.22E-62
**300**	111	3.32E-94	116	1.54E-101	60	4.00E-32	101	3.52E-80	51	7.65E-24	101	3.52E-80
**400**	124*	9.02E-88	168	1.02E-134	86	1.19E-38	149	1.67E-108	71	2.66E-26	145	2.80E-103
												
**IV1 **∩ **IV2**												
**n**	**Colon**		**Kidney**		**Liver**		**Lung**		**Pancreas**		**All**	
	**Overlap**	**P-Value**	**Overlap**	**P-Value**	**Overlap**	**P-Value**	**Overlap**	**P-Value**	**Overlap**	**P-Value**	**Overlap**	**P-Value**
**400**	163*	1.39E-186	355	0	No data	-	144	3.97E-140	No data	-	280	0
												
**SAM1 **∩ **SAM2**												
**n**	**Colon**		**Kidney**		**Liver**		**Lung**		**Pancreas**		**All**	
	**Overlap**	**P-Value**	**Overlap**	**P-Value**	**Overlap**	**P-Value**	**Overlap**	**P-Value**	**Overlap**	**P-Value**	**Overlap**	**P-Value**
**400**	176	1.92E-146	284	0	253	6.86E-281	241	3.15E-257	No data	-	262	2.34E-299

* Only 338 genes are used for colon IV1

**Figure 2:**
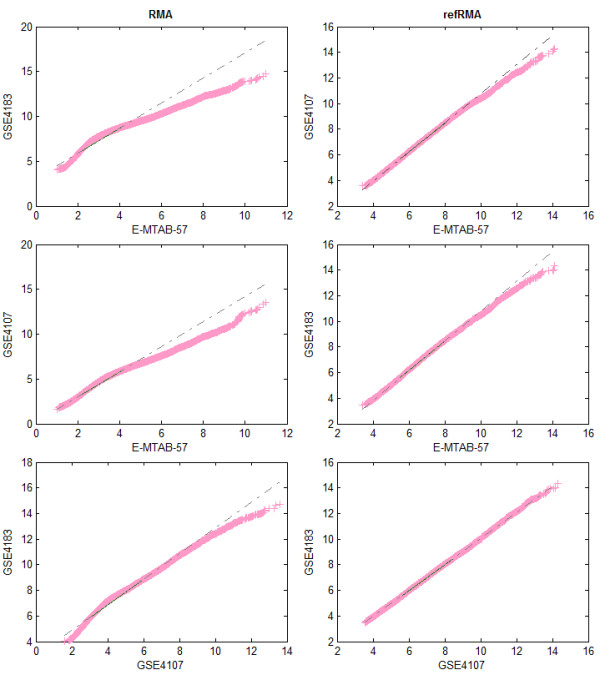
**Quantile-Quantile Plots**: Quantile-quantile plots indicating the distribution of three randomly chosen arrays from different colon datasets based on RMA (left) and refRMA (right) normalization.

Moreover, to assess the effect of the refRMA method in normalizing data, three samples from different colon datasets (E-MTAB-57, GSE4107 and GSE4183) were chosen. The expression values for the three arrays were obtained based on classical RMA and refRMA normalization techniques. Quantile-quantile plots were produced to compare the distributions of the different datasets in a pair-wise manner (Figure 2). The points within the plot should form a straight line if the two arrays have similar distributions. The results in Figure 2 draw attention to the differences in distributions when normalizing datasets individually using RMA as opposed to refRMA's ability to normalize the different datasets to possess similar distributions.

### IV meta-analysis and merged SAM overlap significantly in results

As in previous microarray studies of cancer [21,26-31], the gene lists produced by the two approaches used in this study indicate significant alterations of the transcriptional profile as the tissue is transformed from the normal to the cancer state, with up to thousands of genes possibly undergoing statistically significant expression changes. While the two methods applied to the three dataset groups produced different lists of significant genes for each of the five tissues under consideration, there was a considerable overlap in the results (Table 2). The significance of the intersection between predicted gene lists increased consistently as the number of top-ranked genes used in comparison were increased from 10 to 400. In colon tissue, the overlap with IV1 was confined to 338 significant genes instead of 400, since that was the total number of genes passing the test criteria. At the 400 gene level p-values of the IV1/SAM1 intersection ranged from 2.66E-26 in pancreas to 8.42E-181 in lung, while the most significant overlap in IV1/SAM2 was in kidney (p-value = 1.02E-134). Comparison of the results of the two SAM methods produced even larger commonalities in the gene lists identified. Apart from the colon tissue, there was at least 60% overlap between the top 400 gene-lists generated by the two SAM methods, for any given comparison. The match between the two SAM results became less pronounced with sharp increases in the number of samples added in SAM2. Nevertheless, even with 506 colon cancer samples included in SAM2 as opposed to the 92 used in SAM1, the overlap between the two methods (176 genes) remained significant. The overlap between IV1 and IV2 varied largely among the top ranked 400 genes with a minimum overlap of 144 genes in lung tissue and a maximum overlap of 355 genes in kidney, resulting in vanishing p-values in the latter case (Table 2).

To identify significantly altered genes across the five considered tissue types, the datasets from all tissues were pooled together. Again, SAM2 included additional datasets with cancer or normal samples only. Similarly, the significance of the overlap between the results increased as more top-ranked genes were considered, with p-values equal to 6.82E-97 and 2.80E-103 for the intersection at the top 400 genes level in IV1/SAM1 and IV1/SAM2, respectively (Table 2).

### Cell cycle pathway and mitosis-related cell division biological processes are commonly enriched in cancers

**Figure 3:**
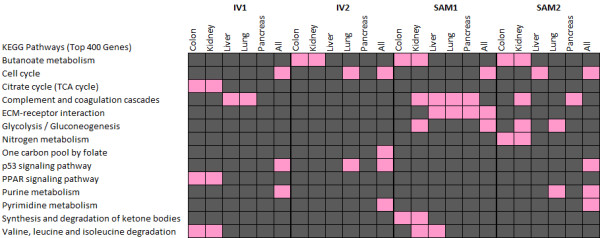
**Enriched KEGG Pathways**: A list of KEGG pathways, shown in pink, that appear to be statistically enriched according to the top 400 genes from IV1, IV2, SAM1 and SAM2 at a p-value cutoff of 0.05. Results are limited to pathways independently enriched in at least two of the tissues or in the combined test including all tissues.

**Figure 4:**
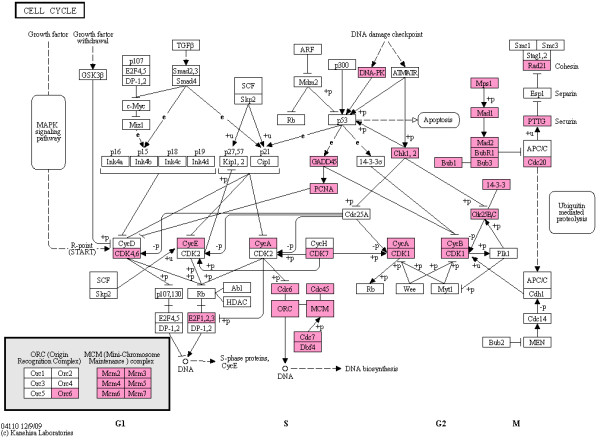
**Cell Cycle Pathway**: Differentially expressed genes involved in the cell cycle are shown in pink. Genes are ranked among the top 400 genes according to at least one of the statistical approaches used (IV1, IV2, SAM1 and/or SAM2), based on analyses of all five tissues together.

The cellular pathways and biological processes that were statistically enriched in the top 400 cancer-associated genes from the multiple tissues under consideration were identified using the DAVID Bioinformatics Resources' [32,33] functional annotation tool as described in the Methods section. Enriched KEGG [34] pathways common to at least 2 tissue types within a given test method or significantly associated with the combined 5-tissue comparisons are shown in Figure 3. The cell cycle pathway was statistically enriched in IV1, IV2, SAM1 and SAM2 gene lists across all tissue types (Figure 4). Among the key changes in the cell cycle in normal to cancer transition are the differential expression of cyclins (A and B) and cyclin-dependent kinases (CDK1 and CDK4/6 complex). CDKs are the core of the regulatory apparatus of the cell cycle progression as changes in the kinases and cyclins drive the cell from one stage of the cell cycle to another [35].

In addition, the p53 signaling pathway and purine metabolism were significantly enriched in all-tissue analyses of both IV tests and SAM2. Pyrimidine metabolism is also enriched for the merged SAM2 significant genes while SAM1 genes are associated with ECM-receptor interaction and glycolysis/gluconeogenesis pathways. At the tissue level, some of the metabolic pathways were common to both kidney and colon cancers (butanoate and nitrogen metabolism). Complement and coagulation cascades were enriched in four out of the five tissues under study. These results show that both methods of integration are capable of reproducing a significant portion of the research literature on cellular pathways activated in cancer.

### Microarray results match cancer research literature with low p-values

**Figure 5:**
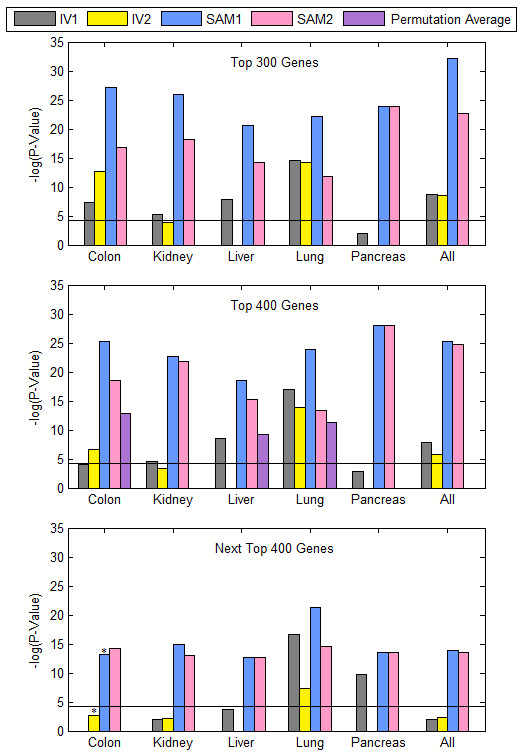
**Literature Search Results**: Histogram representing p-values of the number of top-ranked genes with at least 1 PubMed abstract relating the genes to cancer research from a non-microarray study according to each of the three test procedures. P-values are calculated based on expected data from a hundred random gene lists obtained from the platform and similarly related to non-microarray cancer literature. IV1 results are shown in gray, IV2 in yellow, SAM1 in blue and SAM2 gene lists are in pink. The horizontal line represents a p-value cutoff of 0.0001. * P-values adjusted to maximum number of available top genes.

Next, we tested the SAM1, SAM2, IV1, and IV2 gene lists for PubMed hits associated with cancer. We conducted an automated PubMed abstract search for the genes in the aforementioned lists. All available abstracts in Pubmed were used excluding those that belonged to microarray-based research. Also excluded were abstracts that did not contain the word "cancer". A gene had to have at least one such PubMed abstract match to be considered as a literature search hit. The number of successful hits produced from the merged SAM methods and the IV tests intersected the research literature with significantly higher coverage than would be expected for randomly generated gene lists (Figure 5). The p-values shown in Figure 5 for the top 300 and 400 genes for all three methods were computed by using control gene lists obtained from the same Affymetrix platforms by randomly selecting lists of equal size (300 or 400) and averaging the number of hits over 100 iterations. The p-values for each tissue were then calculated using a normal distribution given the mean and standard deviation parameters of the randomly generated data. The p-value for the colon IV1 in the top 400 gene list was adjusted to a hundred iterations of 338 randomly chosen genes to account for the maximum available number of genes. The merged SAM methods produced gene lists that matched the research literature more accurately than the gene lists produced by the IV tests in four out of the five tissues under consideration. Additional File 1 contains the top 800 gene lists for the cancer types under consideration for SAM1 and IV1 approaches.

PubMed hits on gene lists presented by meta-analysis and merged SAM approaches fell inside and outside the intersections. Consider for example the case of colon cancer in IV1 and SAM1 gene lists. There were 93 hits on IV1 ∩ SAM1 (p = 1.19E-07), 103 hits on IV1 - IV1 ∩ SAM1 (p = 5.09E-02); and 205 hits on SAM1 - IV1 ∩ SAM1 (p = 2.32E-23).

As an additional control, the next top 400 genes (ranks 401-800) in each list, if available, were subjected to a similar PubMed abstract search. The p-values representing the results revealed decreased literature coverage of these genes compared to the first top 400 genes in all cases except for SAM2 results in lung tissue. In this test, majority of the IV results (except for lung and pancreas) dropped below the 0.0001 p-value threshold marked by a horizontal line in the figure.

Our results show that in the merged SAM approach, the more symmetric the datasets are in terms of containing both disease and control samples, the better is the match between gene lists produced by microarray analysis and the PubMed literature. Both SAM1 and SAM2 (containing asymmetric data) produced more significant p-values per tissue than the average p-value obtained from the SAM tests performed on the individual datasets for a given tissue (data not shown). The addition of single sample-type datasets (only cancer or only normal) in SAM2 resulted in fewer literature-associated gene lists than the SAM1 approach; however, the results improved when considering the top 400 genes as opposed to the top 300.

Next we considered an extreme case of SAM2, where the dataset was composed of disease samples from some studies and control samples from other studies. In this case, there would be no symmetric core in the dataset under consideration. To develop such purely asymmetric datasets, we deleted either the disease or the control samples of any symmetric study included in SAM2, considering all possible permutations for the datasets from three tissues: colon, liver and lung. The resulting gene lists were annotated with PubMed hits. We calculated an average number of PubMed hits over all possible combinations and corresponding p-values. The results (shown in Figure 5 with purple bars) produced slightly fewer hits than the original SAM2 approach highlighting the importance of utilizing symmetric datasets when available as the core of the merged SAM technique. Nevertheless, even in this extreme case the probability for the match between literature and microarray gene lists to have occurred by random chance events was extremely small. It is clear from Figure 5 that the merged SAM analysis of purely asymmetric data results in prediction accuracy comparable to meta-analysis utilizing data with disease and control samples coming from the same labs.

## Discussion

Meta-analysis approaches to microarray data aim to increase the statistical power of the results as well as increase reproducibility from individual studies [11]. Typical meta-analysis approaches combine results of independent datasets to produce a generalized outcome across these datasets. Meta-analysis approaches require both perturbed and control data within the same microarray datasets under consideration. However, the recent dramatic increase in public access microarray samples is mainly due to datasets containing no data on normal tissue. Noting that microarray samples on normal tissue are available in other public datastes, we wanted to explore the idea of picking samples from different datasets obtained with same/similar microarray chips and normalizing them together before the identification of significantly altered genes in normal to cancer comparison. The resulting merged SAM sacrifices the use of data from other platforms. However, it could be potentially useful for integrated analysis of cancer microarray datasets for which much of the available data is highly asymmetric. It is important to note that SAM analysis was chosen to determine the significant gene lists since it is believed to be superior to other microarray analysis methods.

A quick study of the GEO database clearly shows that microarray data for hormone-associated solid cancers such as breast, prostate and ovarian cancers are highly asymmetric. The more recent datasets increasingly come from studies for which one cancer subtype is compared to another cancer subtype and as a result contain no data from normal samples. We chose the five tissue types presented in this study because of the availability of data that could be used for both merged SAM and meta-analysis approaches. Previous studies have addressed the possible problems that arise from combining data across different technologies [36,37]. We have used the datasets obtained with similar chips to compare the performance of meta-analysis and merged the SAM approaches. The direct integration of data preceding the analysis as in the case of the merged SAM overcomes the problems associated with small sample sizes in individual studies. While data merging across similar chips sacrifices the inclusion of some of the genes not common to all platforms, it provides additional robustness since all samples are normalized together as opposed to being normalized separately per dataset [38].

We found that meta-analysis and merged SAM approaches yielded significant gene lists with intersecting common gene subsets that could not be plausibly obtained by chance. Both approaches matched automated PubMed abstract searches of research literature (excluding microarray studies) with very low p-values for random occurrence. However, the merged SAM approach replicated the existing literature much more accurately than the meta-analysis approach in five of the six cases under study. Addition of cDNA arrays into meta-analysis resulted in reduced overlap with the cancer literature. Meanwhile, the inclusion of asymmetric datasets also produced slightly less statistically significant results in merged SAM analyses, nevertheless, the approach still generated results that were at least as significant as the meta-analyses, again surpassing meta-analysis in five out of the six cases. Despite the addition of hundreds of samples from asymmetric sets, the merged SAM continued to perform well, matching literature as well as results of symmetric microarray data. We also showed that the match between microarray lists and the literature became less pronounced as lesser-ranked significant genes (401 - 800) were used in the comparison. The gene lists obtained in all the tests were further validated by associating them with functional annotation through KEGG pathways. While individually each tissue possessed a unique list of pathways and processes with which it was associated, overall, cell division appeared to be the common driving factor to all tissues, as would be expected.

We used automated text searches as an instrument for validation of the prediction value of the two different approaches to integrate microarray data associated with cancer. Typical validation used in microarray analysis for illustrating relevance of gene list to disease state under consideration is usually via partitioning the dataset into learning, testing/validation subsets in a supervised learning approach [39-41]. However, it is relatively easy to differentiate between cancer and normal tissue with a variety of gene sets, but in many cases such sets are laboratory specific [42]. Research literature in cancer is rich with data on genes associated with this disease and the bulk of such data was collected by using research tools other than microarrays, and therefore, automated text search constituted an independent means of validating the microarray results. Approximately 520,000 PubMed abstracts were retrieved based on cancer association with genes from the relevant microarray platforms. Among those, 25,000 were associated with cancer but involved microarray studies and were therefore not included in our evaluation. The remaining PubMed hits were used to assign scores to the gene lists obtained and test the significance of these scores.

One reason for asymmetry in the current public access microarray data is that the goals of global gene expression quantification in cancer research shifted towards identifying significant genes associated with cancer subtypes [43-47]. The merged SAM analysis presented here is applicable to any microarray inquiry where there is a perturbed state (say cancer subtype 1) and control state (cancer subtype 2). We chose to illustrate the method of integration with cases where there was plenty of data for both meta-analysis and merged data approaches. Even when one aims to uncover differences in gene expression profile between two cancer subtypes, it is often useful to consider such differences between subtypes and control normal tissue samples [21]. Such triple comparisons reveal the original basis for the subtype differences that stem from normal to cancer transformations.

PubMed hits on gene lists produced by meta-analysis and merged SAM approaches fall on the intersections of such lists as well as outside the intersections, suggesting the use of both approaches whenever data is available. The top ranked 400 genes in both cases are highly statistically enriched with PubMed hits and for which the intersection between the two approaches had typically the lowest p-value. When considering the role of well studied genes such as hub genes or genes in public access cellular pathways, it is straightforward to project both gene lists onto known pathways to generate new hypotheses for experimental verification. The merged SAM technique provides a unique opportunity to obtain a candidate list for genes associated with a perturbed state in cases where the public microarray data is largely asymmetric.

## Conclusions

Typical meta-analysis approaches allow for the use of various platforms at the expense of utilizing large amounts of data that exist in datasets containing either normal or cancer tissues only. Our merged SAM approaches have been shown to reproduce much of the known cancer literature while effectively being applied to asymmetrical microarray datasets. In our merged data approach, SAM analysis could be replaced by other widely used statistical methods, thus increasing the extent of the methodology. Such methods may include both parametric approaches such as PAGE [48] and T-profiler[49], or nonparametric approaches including GSEA [50] and rank products [51], among many others.. While many of the genes in our lists have already been associated with cancer, our approach sheds light on new genes which could play a pivotal role in cancer pathogenesis.

## Methods

### Microarray dataset selection

A total of 31 Affymetrix microarray datasets containing 1,768 unique samples from human cancer (1,429) and corresponding healthy control tissues (339) were collected from the Gene Expression Omnibus (GEO; [2,3] and Array Express [4] online repositories (Additional File 2). Samples were selected for 5 different tissue types: colon, kidney, liver, lung and pancreas, then categorized into cancer and control subsets to allow for intra- and inter-tissue comparisons. The cancer samples were not restricted to a single type of malignancy in order to provide a generalized pathogenic approach shared by cancers. The microarray data were limited to those hybridized on the Affymetrix human microarray platforms HG-U133A, HG-U133A 2.0, and the HG-U133 Plus 2.0, due to the large overlap between the three platforms. In addition, the inclusion criteria restricted that each dataset was obtained from a peer-reviewed study and contained a minimum of 20 usable microarray samples (Figure 1b).

### Normalization and differential expression

For Affymetrix chips, raw microarray CEL files were read using the platform-compatible custom ENTREZG CDF file (version 12) [52] in order to obtain Entrez gene intensities. Where multiple replicates from the same source were available, the gene intensities were averaged across replicates. Nineteen out of 31 datasets contained samples for both the normal and cancer tissues and therefore could be used in meta-analysis. Individual datasets were background adjusted normalized with median polish using the robust multi-array analysis (RMA) in MATLAB [53]. For each tissue, the corresponding log-transformed data were transferred into R [54] and the metaGEM package [11] was utilized to conduct the meta-analysis using inverse variance (IV1). The IV model is based on a relative distance measurement computed as follows:

$$\text{d}(\text{i})=[{\text{x}}_{1}(\text{i})-{\text{x}}_{2}(\text{i})]/\text{s}(\text{i})$$

where x_1_(i) and x_2_(i) are the average levels of gene expression for gene (i) in states 1 and 2, respectively, and s(i) is the gene-specific pooled standard deviation which is equal to:

$$\text{s}(\text{i})=[({\text{n}}_{1}-1){\text{s}}_{1}^{2}+({\text{n}}_{2}-1){\text{s}}_{2}^{2}]/({\text{n}}_{1}+{\text{n}}_{2}-2)$$

where s_1 _and s_2 _are the sample standard deviations of groups 1 and 2, while n_1 _and n_1 _are the number of samples in group 1 and 2, respectively. The false discovery rate (FDR) was set at 0.001%. Moreover, the samr package [55] in R was used to conduct the significance analysis of microarrays (SAM) test [20] on each individual dataset. A hundred permutations were performed and results were restricted to significant genes with an FDR of 0.

While IV analyzes each dataset separately before combining the results, SAM can be applied to previously merged data. This merger was achieved by using the refRMA algorithm [56], designed for large microarray datasets to compute the robust multichip averages. Similar to the classic RMA, background adjustment was applied to each sample from 909-array training set composed of all HG-U133 Plus 2.0 arrays used in this study. Quantile normalization was performed followed by median polishing. The outputs of this training process produces two archived vectors; a probe effect vector compiled from the individual log-scale probe affinity effects and a normalization vector compiled based on the transformed PM intensities. These vectors can then be extended to the samples from the other two platforms by using the predetermined group of arrays to estimate the effects and the average empirical distribution that should be used for the added data. The refRMA model is calculated as follows:

$$\text{T}({\text{PM}}_{\text{ij}})={\text{e}}_{\text{i}}+{\text{a}}_{\text{j}}+\epsilon_{\text{ij}}\text{ such that i}=1,\ldots ,\text{I(arrays) and j}=1,\ldots ,\text{J}(\text{probes})$$

where T is the transformation for the background correction, normalization and log transformation of the perfect match intensities, e_i _is the log_2 _scale expression values of array i, a_j _is the log scale affinity effect of probe j and ε_ij _is the error. A more detailed description can be found in [57].

The genes common to all three platforms are then chosen allowing for the integration of data from all three platforms together, limiting results to the 9,409 genes. To verify the application of refRMA to the added data compared to the original training set, one sample was randomly chosen from each of the three colon Affymetrix datasets that contained both healthy and cancer samples. Quantile-Quantile plots (Q-Q plots) were then generated for these arrays based on their individually normalized values (RMA) and collectively normalized values (refRMA). In each case, all gene expression values from one array are plotted against all gene expression values from the second array in order to assess the similarity in their distributions [14]. A merged SAM test was then applied to the combined data of each tissue using the same datasets included in the IV1 test based on the aforementioned parameters (100 permutations and 0 FDR).

**Figure 6:**
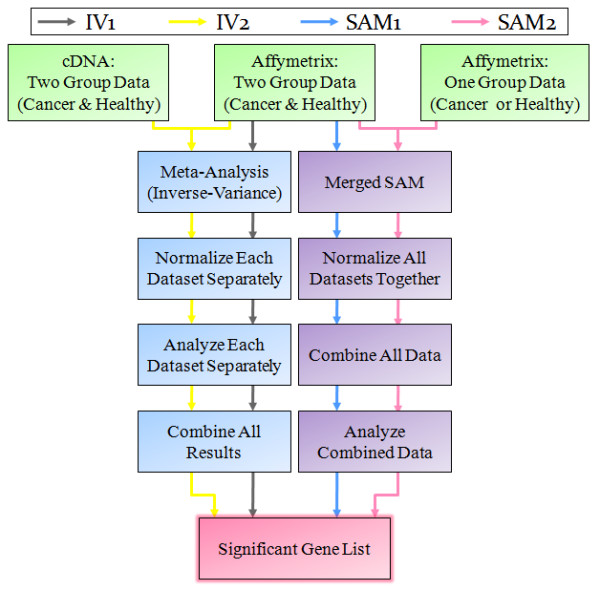
**Workflow of the Analyses**: Flowchart depicting the steps involved in each of the steps involved in each of the four analyses considered: IV1 (grey), IV2 (yellow), SAM1 (blue) and SAM2 (pink).

As noted above, the IV test is limited to datasets that contain both cancer and normal tissues. The merged SAM method, however, allows for the inclusion of datasets containing solely normal or solely cancer samples. Thus, to test the effect of adding such datasets, microarray samples from all datasets of the same tissue were combined together and another series of SAM analyses were applied using the same test parameters as above. For the purpose of this paper, the first set of SAM tests, based on the data from the 19 datasets containing both normal and cancer tissues, is referred to as SAM1 (Figure 6). The second method in which all samples from the 31 datasets could be utilized is denoted as SAM2 (Figure 6). For each tissue, the lists of top 400 differentially expressed genes from the IV and both SAM tests were selected, based on the absolute relative distance measurement in gene expression. These gene lists were used to identify significantly enriched KEGG pathways at a p-value ≤ 0.05 using DAVID Bioinformatics resources [32,33].

### Common transcriptional profiles across all five tissue types

To identify consistent changes that are associated with multiple cancer tissue types, an IV1 test was conducted on all 19 Affymetrix datasets containing both cancer and normal samples together, regardless of tissue type. Similarly, a SAM test was performed on the same samples (SAM1) and another SAM test was applied to all 1,768 available Affymetrix samples from the 5 tissues considered (SAM2; Figure 6). The same test parameters were used as previously mentioned. After determining the genes that behave consistently across all the different cancer types, the top 400 genes were selected from the gene lists produced by each of the methods. Enriched KEGG pathways were identified for all lists at a p-value cutoff of 0.05.

### Expanding IV analysis to cDNA data

An additional 5 datasets using cDNA microarray platforms were obtained from GEO (Additional File 2). These datasets utilized different platforms and the conversion of data to Entrez IDs resulted in the study of varying number of genes per dataset as well as different total overlap with the common Affymetrix platform (shown in parentheses); GSE6988: 9,072 (5,834) genes, GSE3: 12,452 (6,598) genes, GSE7367: 2118 (1,301) genes, GSE2088: 13754 (7,038) genes, and GSE8596: 6740 (4,330) genes. The datasets contained cancer versus normal samples from colon, kidney and lung tissues for a total of 292 cancer and 169 normal samples. No publicly-accessible data could be found for the other two tissues. The IV analyses for these three tissues as well as the combined tissue test were re-run (IV2; Figure 6) to investigate the cost of excluding these datasets from the merged SAM approach that relies solely on Affymetrix data. Similar test parameters were applied, restricting results to genes with an FDR less than 0.001% and top 400 gene lists were utilized for identifying enriched KEGG pathways, as described above.

### Literature verification of results

To determine the extent to which each method replicated the known cancer literature an automated text search was performed. A search of the gene symbol and the term "cancer NOT microarray" was conducted in PubMed abstracts for all genes available from the different platforms, limiting results to non-microarray literature. All gene lists obtained through IV and SAM analyses were then annotated with these results, identifying those genes that were cited in relation to cancer at least once from those that had no cancer association. A hundred random gene lists from the same platform of equal size to the lists under consideration were obtained and used as a control. The number of cancer-related genes in each of these random iteration was determined, and the mean and standard deviation were calculated from these values to obtain the parameters of a normal distribution. The expected value and the standard deviation were then used to compute the p-values for the significant association of each of our cancer gene lists with the known non-microarray literature.

## List of abbreviations used

IV: Inverse Variance; KEGG: Kyoto Encyclopedia of Genes and Genomes; SAM: significance analysis of microarrays.

## Competing interests

The authors declare that they have no competing interests.

## Authors' contributions

ND and AT conceptualized the research. ND implemented the algorithms and discussed results with AT and prepared the first draft of the manuscript. ND and AT both have read and approved the final manuscript.

## Availability

The refRMA model is available by request from [56] and a MATLAB version is available by request from the corresponding author.

## Supplementary Material

Additional file 1 **Top 800 Ranked Genes**: Annotation of the top 800 genes for each tissue according to IV1 and SAM1analyses. Fold changes shown are based on overall values across all platforms and samples. Genes not shared by all three platforms are marked as unique. Click here for file

Additional file 2 **Microarray Samples Used in the Study**: This file contains five worksheets that list all normal and cancer tissue microarray samples used including accession numbers of datasets, sample labels, sample tissue annotation and platform, in addition to malignancy description of cancer samples and available clinical annotation. Click here for file
